# Field Trial Evaluation of the Performances of Point-of-Care Tests for Screening G6PD Deficiency in Cambodia

**DOI:** 10.1371/journal.pone.0116143

**Published:** 2014-12-26

**Authors:** Arantxa Roca-Feltrer, Nimol Khim, Saorin Kim, Sophy Chy, Lydie Canier, Alexandra Kerleguer, Pety Tor, Char Meng Chuor, Sim Kheng, Sovannaroth Siv, Patrick S. Kachur, Walter R. J. Taylor, Jimee Hwang, Didier Menard

**Affiliations:** 1 Malaria Consortium, Phnom Penh, Cambodia; 2 Malaria Molecular Epidemiology Unit, Institut Pasteur in Cambodia, Phnom Penh, Cambodia; 3 Medical Laboratory, Institut Pasteur in Cambodia, Phnom Penh, Cambodia; 4 National Center for Parasitology, Entomology and Malaria Control (CNM), Phnom Penh, Cambodia; 5 U.S. Centers for Disease Control and Prevention, Atlanta, Georgia, United States of America; 6 Centre de Médecine Humanitaire, Hôpitaux Universitaires de Genève, Geneva, Switzerland; 7 Global Health Group, University of California San Francisco, San Francisco, California, United States of America; Quensland University of Technology, Australia

## Abstract

**Background:**

User-friendly, accurate, point-of-care rapid tests to detect glucose-6-phosphate dehydrogenase deficiency (G6PDd) are urgently needed at peripheral level to safely recommend primaquine for malaria elimination.

**Methods:**

The CareStart G6PD RDT (AccessBio, New Jersey, USA), a novel rapid diagnostic test and the most commonly used test, the fluorescent spot test (FST) were assessed against the quantitatively measured G6PD enzyme activity for detecting G6PDd. Subjects were healthy males and non-pregnant females aged 18 years or older residing in six villages in Pailin Province, western Cambodia.

**Findings:**

Of the 938 subjects recruited, 74 (7.9%) were severe and moderately severe G6PD deficient (enzyme activity <30%), mostly in male population; population median G6PD activity was 12.0 UI/g Hb. The performances of the CareStart G6PD RDT and the FST, according to different cut-off values used to define G6PDd were very similar. For the detection of severe and moderately severe G6PDd (enzyme activity <30%, <3.6 UI/g Hb) in males and females, sensitivity and negative (normal status) predictive value were 100% for both point-of-care tools. When the G6PDd cut-off value increased (from <40% to <60%), the sensitivity for both PoCs decreased: 93.3% to 71.7% (CareStart G6PD RDT, p = 10^−6^) and 95.5% to 73.2% (FST, p = 10^−6^) while the specificity for both PoCs remained similar: 97.4% to 98.3% (CareStart G6PD RDT, p = 0.23) and 98.7% to 99.6% (FST, p = 0.06). The cut-off values for classifying individuals as normal were 4.0 UI/g Hb and 4.3 UI/g Hb for the CareStart G6PD RDT and the FST, respectively.

**Conclusions:**

The CareStart G6PD RDT reliably detected moderate and severe G6PD deficient individuals (enzyme activity <30%), suggesting that this novel point-of-care is a promising tool for tailoring appropriate primaquine treatment for malaria elimination by excluding individuals with severe G6PDd for primaquine treatment.

## Introduction

Primaquine (PQ), an 8-aminoquinoline, is the only available and effective drug both to block *P. falciparum* transmission by killing mature gametocytes and to prevent relapses of the persistent liver stage of *P. vivax* [1]. Recently, the World Health Organization (WHO) has recommended, without prior G6PDd testing, the routine addition of single, low-dose PQ (0.25 mg base/kg) as a gametocytocide for *P. falciparum* infected patients, especially in areas of artemisinin resistance [2], reassuring countries including Cambodia who have been reluctant to adopt this policy without additional safety data. Currently, the main limitation to the use of primaquine, for the radical cure of *P. vivax* or *P. ovale* malaria, is the risk of haemolysis in patients who are glucose 6-phosphate dehydrogenase (G6PD) deficient [1], [2]. High doses of PQ are rarely used in malaria endemic countries mainly due to concerns of potentially fatal. Cambodia does not use PQ as antirelapse treatment for vivax malaria because the high dose involved (30 mg base daily) would cause significant haemolytic toxicity if given wrongly to G6PDd patients [3]–[6]. Thus, the availability of an inexpensive point-of-care (PoC) test could have tremendous impact on vivax elimination.

G6PDd is an X-linked, hereditary genetic defect due to mutations in the *glucose-6-phosphate dehydrogenase* gene, which cause functional variants with many biochemical/clinical phenotypes [7]. G6PD, the key enzyme in the oxidative pentose phosphate pathway, converts nicotinamide adenine dinucleotide phosphate (NADP) into its reduced form, NADPH. NADPH is essential for protecting erythrocytes against oxidative stress, by reduction of glutathione disulfide. G6PDd causes increased susceptibility of erythrocytes to reactive oxygen species that can lead to hemolytic anemia [7]. Drugs such as PQ and Dapsone, fava beans and infections can induce hemolytic anemia in G6PD-deficient individuals [8]. In Cambodia, severe and moderate G6PDd are frequent (14.0%) [9]–[12], higher in western (15.3%) compared to eastern populations (8.9%) [11]. ViangChan is the most common variant (WHO class II variant), accounting for more than 90% of G6PDd cases. Its median G6PD enzyme activity is 0.8 UI/g Hb, representing 0.7% of the population [11], [12].

G6PDd can be detected reliably in homozygous women and hemizygous males with a number of tests. In heterozygous women, diagnosing G6PDd is more difficult, and a large part of this group is usually missed by the standard tests [13]. Indeed, heterozygote females with mixed G6PD normal and deficient red cells, display varying levels of enzyme deficiency due to the partial inactivation of one X chromosome, a phenomenon known as lyonisation [14]. To date, the ‘gold standard’ diagnostic method of G6PDd is based on the estimation of enzyme activity, by quantitative spectrophotometric analysis of the rate of NADPH production from NADP+ [7], [15]. For rapid population screening and for guiding case management, several semi-quantitative methods are in use, [16]–[21] but most of them, including the most commonly used test, the fluorescent spot test (FST), are costly, time-consuming, and requires additional equipment and specified storage conditions, thus, limiting its use in the field [22], [23].

In 2011, a rapid diagnostic test (RDT)-format test (CareStart, first generation G6PDd screening test, Access Bio, New Jersey, USA) was evaluated by comparing its performance to measure G6PD enzyme activity [12]. Its sensitivity and specificity were estimated to be 68% (66/97) and 100% (806/806), respectively. Although its detection threshold was noted to be 2.7 UI/g Hb, well within the range of moderate and severe enzyme deficiencies, thirteen subjects (1.4%, 12 males and 1 female) with G6PD enzyme activities below 2.0 UI/g Hb were falsely classified as normal. As these patients would potentially have received PQ, they would have been at risk of hemolysis. Recently, a novel (third generation) CareStart G6PD RDT has been recently developed by the same manufacturer.

In the study presented, here, we assessed under field conditions, in a population-based cross- sectional survey, this novel RDT-format test against the G6PD quantitative assay (‘gold standard’) and the FST (‘clinical standard’), as a potential alternative tool to guide PQ treatment for the radical cure of *P. vivax*.

## Methods

### Study site and population

The study took place in Pailin Province, western Cambodia, area with high rates of G6PDd (∼18%) [11] in six villages (Andong Buon, Bar Yakha, Krachab Kroam, Oh Preus, Pang Roluem and Prey MangKol). The inclusion criteria were individuals aged ≥ 18 years who were competent to give informed consent. Pregnant and lactating women were excluded. The sample size was calculated using the precision method to observe an expected sensitivity and specificity of ≥90% with a 5% precision. Assuming a 15% G6PD prevalence, >900 participants needed to be recruited to obtain an expected number >135 G6PDd subjects.

### Field procedures and sample collection

In the selected villages, all inhabitants providing informed consent were included in the study, as previously performed [12]. After obtaining informed consent, a brief questionnaire was completed and the axillary temperature was recorded. Individuals with ≥ 37.5°C were tested for malaria using a RDT (G0131, CareStart Malaria HRP2/pLDH, Pf/PAN, Access Bio, New Jersey, USA) and treated with Duo-Cotecxin (dihydroartemisinin plus piperaquine combination, Zhejiang Holley Nanhu, Pharamaceutical Co. Ltd, Jiaxing City, China) as recommended in the Cambodian National Treatment Guidelines, if testing was positive.

Blood was collected both by finger prick (25 µL) and by venous puncture (2 mL in an EDTA tube). The capillary blood sample was used to perform the CareStart G6PD RDT (Access Bio, New Jersey, USA) and the FST (ref 203-A, Trinity Biotech, St. Louis, USA), according to manufacturers' instructions. Briefly, for the CareStart G6PD RDT, two microliters of blood were collected using the lancet provided in the kit and added into the sample well and two drops of buffer into the buffer well. Test results were read visually after 10 minutes (using a watch). Samples with normal G6PD activity produce a distinct purple color background in the result window while no color change was observed at the test read time for samples with deficient G6PD activity. Samples with a pale purple color background were conservatively classified as deficient. For both PoC, readings were made by two independent blinded researchers. When discordances were recorded between the two readers (result reported as normal by one reader and deficient by the second reader), the final decision was to consider the individual as deficient. Venous blood samples were stored at 4°C in cool boxes and transported to Phnom Penh within 24 hours. Before their use in the field, the validity of both PoCs was monitored by three levels of G6PD controls (deficient, intermediate and normal, Trinity Biotech, St. Louis, USA).

### Laboratory procedures

The cells blood count (CBC), the determination of the G6PD enzyme activity and the capillary Hb electrophoresis was done as previously described [11]. On a random sub-set of samples, capillary and venous blood were assessed for concordance on the CareStart G6PD RDT (n = 48) and the FST (n = 38). Venous blood and white cell depleted blood using a CF11 column [24] was also assessed for concordance (n = 31 for CareStart G6PD RDT and n = 38 for FST) to explore if leukocytes and platelets which may be rich in G6PD enzyme cause some interference in the assays, as has been already observed [25].

DNA was extracted from red blood cells and the buffy coat in all individuals with < 60% of normal G6PD enzyme activity using the QIAamp DNA Blood Mini Kit (Qiagen, Courtaboeuf, France), according to the manufacturer's instructions. They were used to perform the detection of malaria parasites by PCR [26] and to detect mutations in the *glucose-6-phosphate dehydrogenase* gene [12], respectively.

### G6PDd detection and haemoglobinopathies classification

The normal value of G6PD enzymatic activity in Cambodian adults was defined by using the adjusted median (100% G6PD activity), as previously described [27]. The adjusted median value was determined from the G6PD enzyme activity values measured in the male population, after excluding males with severe G6PD deficiency (G6PD enzyme activity < 10% normal). G6PDd was defined as a percentage of normal G6PD activity and individuals were classified according to different cut off values of residual activity (<10%, <20%, <30%, <40%, <50%, <60%, ≥60%).

Normal haemoglobin profile and haemoglobinopathies were defined as previously described [11].

### Data management and statistical analyses

Data were recorded on a case reporting form (CRF), double-entered into an electronic database and analyzed using MedCalc software (version 9.1.0.1; Mariakerke, Belgium).

According to the different cut-offs used to define G6PDd and using the quantitative G6PD assay as the gold standard, classical diagnostic test performance measures were determined: Sensitivity (Se), Specificity (Sp), Deficient/Positive Predictive Value (PPV) and Normal/Negative Predictive Value (NPV) [28]. Test performances of PoCs were also compared between samples collected from capillary/venous blood, and from capillary/white cell depleted venous blood.

The mean, median, standard deviation and ranges were determined for all G6PD enzyme activity values by gender and type of blood sample (capillary, venous, white cell depleted venous). The ANOVA test, the Mann-Whitney *U* test and the Student's t test were used for comparisons of continuous variables. For categorical variables, Chi-squared or Fisher's exact tests were used to assess significant differences in proportions. Odds ratios were used to compare the relative odds of the occurrence of G6PDd, given exposure to the variable of interest [29]. Comparisons showing p-value < 0.05 was considered to be statistically significant.

### Ethical considerations

The study protocol was reviewed and approved by the National Ethics Committee for Health Research of the Ministry of Health of Cambodia (approval number 275 NECHR). The U.S. Centers for Disease Control and Prevention Institutional Review Board reviewed and granted non-engaged status. Informed written consent was provided by all individuals before inclusion in the study and all investigations were conducted according to the principles expressed in the Declaration of Helsinki. Results for each patient (according to the quantitative G6PD activity test) were given to the local Ministry of Health staff of Pailin province involved in the study.

## Results

### Study population and distribution of the G6PD enzymatic activity

From February-May 2013, 938 subjects were enrolled from six villages in Pailin Province. The male/female ratio was 451/487 (0.93) and age ranged from 18 to 75 years old (median = 32 years, IQR 24–48 years) (Table 1). All participants declared they were ethnic Khmer. Approximately, one third had an abnormal haemoglobin electrophoresis profile and 13.6% (127/938) had <60% of normal enzyme activity, according to the quantitative G6PD activity test. No significant differences were observed between villages, except for age, white and red blood cells counts and haemoglobin electrophoresis profiles. Among 19 febrile patients, 3 were found positive by RDT and treated with Duo-Cotecxin (Table 1). The proportion of positive parasite carriers detected by PCR was 1.9% (14/750). *P. vivax* was the most prevalent species (12/14, 86%). Only one individual with *P. vivax* infection had a 30–40% of normal G6PD enzyme activity. The other malaria cases were G6PD normal including two falciparum malaria cases. No significant differences were observed in G6PD enzyme activity median between malaria-infected and non-infected individuals (12.2 *vs*. 11.7 UI/g Hb, p = 0.70, Mann-Whitney *U* test).

**Table 1:** Characteristics of the study population, Pailin, Cambodia, 2013.

Patients' characteristics		Andong Buon	Bar Yakha	Krachab Kroam	Ou Preus	Pang Roluem	Prey Mangkol	Total	p-value ø
**No. of enrolled individuals**		89	80	95	208	247	219	938	-
**Median age in years (range)**		31 (18–75)	33 (18–67)	40 (18–63)	40 (18–75)	25 (18–74)	32 (18–75)	32 (18–75)	<10^−6^*
**Sex ratio (% female)**		47	59	59	52	52	48	52	0.38 ^**^
**No. Individuals with T°> 37.5°C (%)**		3 (3.5)	2 (2.6)	0	4 (2.0)	5 (2.4)	5 (2.3)	19 (2.1)	0.16 ^**^
**Malaria: No. of individuals found positive by**	RDT*	0/3	0/2	-	0/4	3/5 (1 Pf/non-Pf and 2 Non-Pf)	0/5	3/19	**-**
	**PCR**	3/89 (1 Pf, 2 Pv)	0/80	0/88**	8/208 (8 Pv)	1/66 (1 Pv)***	2/219 (1 Pf, 1 Pv)	14/750	**-**
**Median WBC - ×10^−3^/mm^3^ (IQR)**		6.9 (5.8–8.2)	6.8 (5.8–7.9)	7.2 (6.3–8.6)	6.4 (5.5–7.6)	7.0 (6.1–8.4)	7.0 (5.9–8.6)	7.1 (5.7–8.2)	<0.0005 *
**Median RBC - ×10^−6^/mm^3^ (IQR)**		4.3 (3.9–4.6)	4.2 (3.9–4.5)	4.0 (3.6–4.4)	4.1 (3.9–4.6)	4.2 (3.9–4.6)	4.3 (4.0–4.6)	4.2 (3.9–4.6)	0.01 *
**Median haemoglobin in g/dL (95% CI)**		11.3 (10.4–12.4)	11.1 (10.3–12.4)	11.2 (10.2–12.1)	11.4 (10.4–12.5)	11.1 (10.4–12.3)	11.5 (10.6–12.8)	11.5 (10.6–12.3)	0.08 *
**Haemoglobin electrophoresis profile (%)**	**Normal**	56 (64.0%)	45 (56.0%)	62 (65.0%)	146 (70.0%)	148 (60.0%)	143 (65.0%)	600 (64.0%)	<10^−6 **^
	**Hb E - homozygous**	0	0	0	0	9 (4.0%)	0	9 (1.0%)	
	**Hb E - heterozygous**	0	0	27 (29.0%)	40 (19.5%)	79 (31.5%)	23 (10.5%)	169 (18.0%)	
	**Heterozygous E - α-thalassaemia**	28 (31.0%)	28 (35.0%)	0	7 (3.0%)	1 (0.5%)	31 (14.5%)	95 (10.0%)	
	**Heterozygous Hb E - β-thalassaemia**	4 (4.0%)	5 (6.0%)	4 (4.0%)	12 (6.0%)	0	14 (6.0%)	39 (4.0%)	
	**α-thalassaemia**	1 (1.0%)	2 (3.0%)	1 (1.0%)	2 (1.0%)	10 (4.0%)	2 (1.0%)	18 (2.0%)	
	**β-thalassaemia**	0	0	1 (1.0%)	1 (0.5%)	0	6 (3.0%)	8 (1.0%)	
**G6PD activity in UI/g Hb**	**Range**	0.9–42.5	0.4–24.5	0.6–21.6	0.5–24.3	0.3–27.6	0.3–45.9	0.3–45.9	0.08 *
	**Mean (95% CI)**	12.2 (10.9–13.4)	11.2 (10.2–12.3)	12.1 (11.3–12.9)	11.4 (10.8–11.9)	10.9 (10.3–11.4)	11.9 (11.2–12.6)	11.5 (11.2–11.8)	
	**Median (IQR)**	12.6 (10.1–14.6)	12.3 (10.2–13.6)	12.1 (10.6–14.2)	11.4 (10.0–12.9)	11.6 (9.0–13.5)	11.9 (10.3–13.8)	11.8 (10.1–13.7)	

* only individuals with T° > 37.5°C were tested by RDT (total  =  19).

All individuals were tested from malaria by PCR (total  =  750), except ** for isolates from Krachab Kroam village (88/95 tested isolates) and *** for isolates from Pang Roluem village (66/247 tested isolates).

ø comparison between villages.

* ANOVA test, ** Chi-squared test.

### Reference range of G6PD enzymatic activity, prevalence of G6PDd and G6PD variants

**Figure 1:**
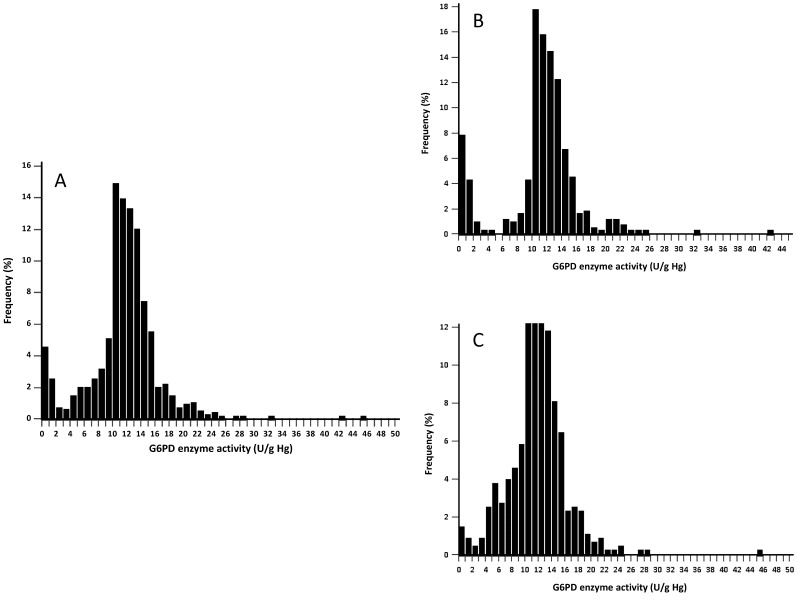
Distribution of the G6PD enzymatic activity (UI/g Hb) values according to gender, of 938 Cambodians adults residing in six villages of the Pailin province, Cambodia, 2013. (Panel A: Total population, Panel B: Male population and Panel C: Female population).

G6PD enzymatic activity values ranged from 0.3 to 45.9 UI/g Hb (Fig. 1, Table 2). No significant difference for median was observed between gender (p = 0.31, Mann-Whitney *U* test). Based on the adjusted male median, 100% G6PD activity was estimated to be 12.0 UI/g Hb and cut-off values were defined as presented in Table 3. The prevalence of G6PDd was as follows: 56/938 (6.0%) with <10% of normal G6PD enzyme activity; 68/938 (7.2%) with <20% of normal G6PD enzyme activity; 74/938 (7.9%) with <30% of normal G6PD enzyme activity; 89/938 (9.5%) with <40% of normal G6PD enzyme activity; 107/938 (11.4%) with <50% of normal G6PD enzyme activity; 127/938 (13.5%) with <60% of normal G6PD enzyme activity.

**Table 2:** Reference values of the G6PD enzymatic activity profile for the study population, Pailin, Cambodia, 2013.

Reference values	Female	Male	Adjusted male*
**Number of cases**	487	451	**392**
**Mean (95% CI) UI/g Hb**	11.8 (11.4–12.2)	11.2 (10.7–11.7)	**12.8 (12.4–13.1)**
**SD**	4.4	5.1	**3.3**
**Median (95% CI) UI/g Hb**	11.9	11.7	**12.0**
**IQR**	9.7–14.0	10.2–13.4	**10.6–13.6**
**Range**	0.5–45.9	0.3–42.5	**7.4–42.5**

* Adjusted median (100% G6PD activity): an adjusted median value is calculated for which males with severe G6PD deficiency (activity less than 10% normal) have been excluded. This is accomplished by:1. Exclusion of all males with G6PD activity equal to or less than 10% of the male median; 2. Determination of a new median G6PD activity [27].

**Table 3:** Diagnostic test performances of the CareStart G6PD RDT and the fluorescent spot test, according to the cut-off values used to define G6PDd, Pailin, Cambodia, 2013.

**Cut-off values**	**in %**	<10%	<20%	<30%	<40%	<50%	<60%	≥60%
	**in UI/g Hb**	<1.2	<2.4	<3.6	<4.8	<6.0	<7.2	≥7.2
**No. of samples according to the cut-off values**		**56**	**68**	**74**	**89**	**107**	**127**	**811**
**Fluorescent spot test**	**No. of individuals classified as deficient/intermediate**	56	68	74	85	90	93	3
	**No. of individuals classified as normal**	0	0	0	4	17	34	808
	**Sensitivity (95% CI)**	100% (93.6–100%)	100% (94.7–100%)	100% (95.1–100%)	95.5% (88.9–98.8%)	84.1% (75.8–90.4%)	73.2% (64.6–80.7%)	
	**Specificity (95% CI)**	95.7% (93.9–96.7%)	96.8% (95.4–97.9%)	97.5% (96.1–98.4%)	98.7% (97.7–99.4%)	99.3% (98.4–99.7%)	99.6% (98.9–99.9%)	
	**Deficiency status Predictive Value - PPV (95% CI)**	58.3% (47.8–68.3%)	70.8% (60.7–79.7%)	77.1% (67.3–85.1%)	88.5% (80.4–94.2%)	93.8% (86.9–97.7%)	96.9% (91.1–99.4%)	
	**Normal status Predictive Value - NPV (95% CI)**	100% (99.6–100%)	100% (99.6–100%)	100% (99.6–100%)	99.5% (98.8–99.9%)	98.0% (96.8–98.8%)	96.0% (94.4–97.2%)	
**CareStart G6PD RDT**	**No. of individuals classified as deficient/intermediate**	56	68	74	83	88	91	14
	**No. of individuals classified as normal**	0	0	0	6	19	36	797
	**Sensitivity (95% CI)**	100% (93.6–100%)	100% (94.7–100%)	100% (95.1–100%)	93.3% (85.9–97.5%)	82.2% (73.7–89.0%)	71.7% (63.0–79.3%)	
	**Specificity (95% CI)**	94.4% (92.7–95.9%)	95.8% (94.2–97.0%)	96.4% (95.0–97.6%)	97.4% (96.1–98.4%)	98.0% (96.7–98.8%)	98.3% (97.1–99.1%)	
	**Deficiency status Predictive Value - PPV (95% CI)**	53.3% (43.3–63.1%)	64.8% (54.8–73.8%)	70.5% (60.8–79.0%)	79.1% (70.0–86.4%)	83.8% (75.3–90.3%)	86.7% (78.6–92.5%)	
	**Normal status Predictive Value - NPV (95% CI)**	100% (99.6–100%)	100% (99.6–100%)	100% (99.6–100%)	99.3% (98.4–99.7%)	97.7% (96.5–98.6%)	95.7% (94.1–97.0)	

Males were more frequently severely deficient: <10% of normal G6PD enzyme activity (48/451 in males *vs.* 8/487 in females, OR = 6.4, 95% CI: 3.0–13.8, p<10^−7^, Fisher's exact test), <20% of normal G6PD enzyme activity (57/451 in males *vs.* 11/487 in females, OR = 5.6, 95% CI: 2.9–10.8, p<10^−8^, Fisher's exact test), <30% of normal G6PD enzyme activity (59/451 in males *vs.* 15/487 in females, OR = 4.2, 95% CI: 2.4–7.6, p<10^−7^, Fisher's exact test) and <40% of normal G6PD enzyme activity (59/451 in males *vs.* 30/487 in females, OR = 2.1, 95% CI: 1.3–3.4, p<10^−3^, Fisher's exact test).

Among the 127 G6PDd individuals (<60% of normal G6PD enzyme activity), sequencing of the G6PD gene detected 6 G6PD variants (Table 4). The most prevalent was G6PD-ViangChan (117/127, 92.0%,) followed by G6PD-Canton (4/127, 3.2%), G6PD-CoImbra and G6PD-Mediteranean (2/127, 1.6% and 2/127, 1.6%, respectively) and G6PD Chinese-5 and G6PD-Mahidol (1/127, 0.8%, and 1/127, 0.8%, respectively).

**Table 4:** G6PD enzymatic activity by G6PD-variants in hemizygous males, homozygous and heterozygous females (n = 127), Pailin, Cambodia, 2013.

Variants	Exon	Mutation	G6PD enzyme activity (UI/g Hb)	Hemizygous male	Homozygous female	Heterozygous female	Total
**ViangChan**	9	871G>A, 1311C>T, IVS11 nt93T>C	N	55	15	49	117 (92.1%)
			Range	0.3–3.2	0.5–3.2	3.9–7.1	
			Mean (95% CI)	1.0 (0.8–1.1)	1.5 (1.0–2.0)	5.6 (5.3–5.9)	
			Median (IQR)	0.9 (0.6–1.2)	1.2 (0.8–2.4)	5.6 (4.6–6.4)	
**Mediterranean**	6	563C>T	N	2	0	0	2 (1.6%)
			Range	-	-	-	
			Mean (95% CI)	0.4	-	-	
			Median (IQR)	0.4	-	-	
**Canton**	12	1376G>T	N	1	0	3	4 (3.1%)
			Range	-	-	5.5–7.1	
			Mean (95% CI)	0.8	-	6.3 (4.3–8.3)	
			Median (IQR)	0.8	-	6.4 (5.7–6.9)	
**CoImbra**	6	592C>T	N	1	0	1	2 (1.6%)
			Range	-	-	-	
			Mean (95% CI)	0.5	-	4.3	
			Median (IQR)	0.5	-	4.3	
**Mahidol**	6	487G>A	N	0	0	1	1 (0.8%)
			Range	-	-	-	
			Mean (95% CI)	-	-	4.7	
			Median (IQR)	-	-	4.7	
**Chinese-5**	9	1024C>T	N	0	0	1	1 (0.8%
			Range	-	-	-	
			Mean (95% CI)	-	-	4.5	
			Median (IQR)	-	-	4.5	

### Performances of PoC tests to detect G6PDd

Defining G6PDd as G6PD activity <60% (n = 127), the sensitivity, specificity, PPV and NPV for the CareStart G6PD RDT were 71.7% (91/127), 98.3% (797/811), 86.7% (91/105) and 95.7% (797/833). Corresponding FST values were: 73.3% (93/127), 99.6% (808/811), 96.9% (93/96) and 96.0% (808/842).

**Figure 2:**
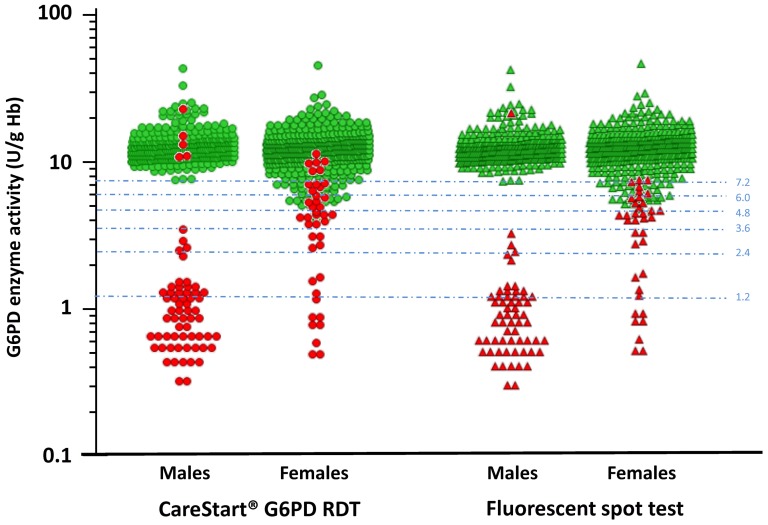
Distribution of the G6PD enzymatic activity (UI/g Hb) values by gender according to point-of-care tests results in 938 Cambodians adults, Cambodia, 2013. CareStart G6PD RDT: green circle (G6PD-normal) and red circle (G6PD-deficient); fluorescent spot test: green triangle (G6PD-normal) and red triangle (G6PD-deficient). The cut-off values (from 10% to 60%) used to define G6PDd are presented as pale blue dash lines.

The performances of the CareStart G6PD RDT and the FST, according to the cut-off values used to define G6PDd were very similar (Table 3). No false normal results were observed in G6PD deficient individuals defined with a cut-off value <30% (<3.6 UI/g Hb). When the G6PDd cut-off value increased (from <40% to <60%), the sensitivity for both PoCs decreased: 93.3% to 71.7% (CareStart G6PD RDT, p = 10^−6^, Fisher's exact test) and 95.5% to 73.2% (FST, p = 10^−6^, Fisher's exact test) while the specificity for both PoCs remained similar: 97.4% to 98.3% (CareStart G6PD RDT, p = 0.23, Fisher's exact test) and 98.7% to 99.6% (FST, p = 0.06, Fisher's exact test). The cut-off values for classifying individuals as normal were 4.0 UI/g Hb and 4.3 UI/g Hb for the CareStart G6PD RDT and the FST, respectively (Fig. 2).

In the male population, the CareStart G6PD RDT had a 100% sensitivity and NPV, for detecting G6PDd, regardless the cut-off values used to define G6PDd. Only five males (5/392, 1.3%) with a G6PD enzyme activity ≥60% were misclassified as G6PD deficient. Amongst females, the CareStart G6PD RDT had a 100% sensitivity and NPV at G6PD enzyme activities <30% (<3.6 UI/g Hb). Thereafter, the sensitivity declined markedly with increasing G6PD enzyme activity. The proportion of females classified as G6PD normal increased with the cut-off values: 6/15 (40%) at threshold <40% (<4.8 UI/g Hb); 13/18 (72%), <50% (<6.0 I/g Hb); 17/20 (85%), <60% (<7.2 UI/g Hb) and 410/419 (97.8%), ≥60% (≥ 7.2 UI/g Hb) (Fig. 2 and Table 5).

**Table 5:** Diagnostic test performances of the CareStart G6PD RDT by gender, according to the cut-off values used to define G6PDd, Pailin, Cambodia, 2013.

Cut-off values	in %	<10%	<20%	<30%	<40%	<50%	<60%	≥60%
	in UI/g Hg	<1.2	<2.4	<3.6	<4.8	<6.0	<7.2	≥7.2
**Males**								
**No. of samples classified as G6PD deficient according to the cut-off value**		**48**	**57**	**59**	**59**	**59**	**59**	**392**
**CareStart G6PD RDT**	**No. of males classified as deficient/intermediate**	48	57	59	59	59	59	5
	**No. of males classified as normal**	0	0	0	0	0	0	387
	**Sensitivity (95% CI)**	100% (92.6–100%)	100% (93.7–100%)	100% (93.9–100%)	100% (93.9–100%)	100% (93.9–100%)	100% (93.9–100%)	
	**Specificity (95% CI)**	96.0% (93.6–97.7%)	98.2% (96.3–99.3%)	98.7% (97.1–99.6%)	98.7% (97.1–99.6%)	98.7% (97.1–99.6%)	98.7% (97.1–99.6%)	
	**Deficiency status Predictive Value - PPV (95% CI)**	75.0% (62.6–85.0%)	89.1% (78.7–95.5%)	92.2% (82.7–97.4%)	92.2% (82.7–97.4%)	92.2% (82.7–97.4%)	92.2% (82.7–97.4%)	
	**Normal status Predictive Value - NPV (95% CI)**	100% (99.1–100%)	100% (99.1–100%)	100% (99.1–100%)	100% (99.1–100%)	100% (99.1–100%)	100% (99.1–100%)	
**Females**								
**No. of samples classified as G6PD deficient according to the cut-off value (%)**		**8**	**11**	**15**	**30**	**48**	**68**	**419**
**CareStart G6PD RDT**	**No. of females classified as deficient/intermediate**	8	11	15	24	29	32	9
	**No. of females classified as normal**	0	0	0	6	19	36	410
	**Sensitivity (95% CI)**	100% (63.1–100%)	100% (71.5–100%)	100% (78.2–100%)	80.0% (61.4–92.3%)	60.4% (45.3–74.2%)	47.0% (34.6–59.6%)	
	**Specificity (95% CI)**	93.1% (90.4–95.2%)	95.5% (93.2–97.2%)	94.5% (92.0–96.4%)	96.2% (96.5–99.2%)	97.3% (95.3–98.6%)	97.9% (95.9–99.0%)	
	**Deficiency status Predictive Value - PPV (95% CI)**	19.1% (8.8–34.9%)	34.4% (18.6–53.2%)	36.6% (22.1–53.1%)	75.0% (56.6–88.5%)	70.7% (54.5–83.9%)	78.0% (62.4–89.4%)	
	**Normal status Predictive Value - NPV (95% CI)**	100% (99.2–100%)	100% (99.2–100%)	100% (99.2–100%)	98.6% (97.1–99.5%)	95.7% (93.4–97.4%)	91.9% (89.0–94.3%)	

### Impact of the blood source (capillary *versus* venous blood) and the white blood cells (WBC)

The results of the CareStart G6PD RDT (n = 48) performed in parallel by using capillary and venous blood were concordant in 47/48 (98.0%): 24 capillary/venous blood samples and 23 capillary/venous blood samples were both classified as G6PD deficient and G6PD normal, respectively. The finger prick sample of one female (aged 34 years, G6PD enzyme activity 6.4 UI/g Hb, ViangChan variant) provided a G6PD normal result while the venous blood a G6PD deficient result. Among the 38 individuals tested in parallel with the FST all samples were concordant (100%, 20 deficient and 18 normal).

The results of the CareStart G6PD RDT (n = 31) performed in parallel by using fresh venous blood before and after WBC removal were concordant in 30/31 (97.0%, 14 normal and 16 deficient). For one female (aged 24 years, G6PD enzyme activity 8.1 UI/g Hb, WBC 4.6 ×10^−3^/mm^3^), her fresh venous blood provided a G6PD normal result while her venous blood after WBC removal, a G6PD deficient result. Among the 38 individuals tested in parallel with the FST, all samples were concordant (100%, 20 deficient and 18 normal).

## Discussion

Results from this study provide strong evidence on the ability of the CareStart G6PD RDT to detect reliably G6PD deficient individuals in males and in females with enzyme activity levels <30% of normal G6PD enzyme activity (<3.6 UI/g Hb), with performance properties comparable to the commonly used FST. The CareStart G6PD RDT therefore represents an excellent and inexpensive (cost/test ∼ $1.50 US) alternative PoC to the FST especially considering FST's costs, time to result, additional equipment requirements and specified storage conditions [22], [23]. Even, the sensitivity of the CareStart G6PD RDT for diagnosing G6PDd defined as <60% of normal G6PD enzyme activity) was modest (∼70%), the CareStart G6PD RDT was 100% sensitive and specific for diagnosing all severe, and moderately severe G6PDd (<30% enzyme activity). In this group, no false normal was recorded. This is a considerable improvement on the previous generation test, which demonstrated lower sensitivity and unacceptably high risk of diagnosing false ‘normal’ status to several severe class II variants [12].

The reason for testing for patients for G6PDd is to identify those who may be at risk of PQ induced haemolysis. This risk is greatest in those with lower G6PD enzyme activities who will receive PQ for radical cure [3], [30]–[32]. The current recommended dose for vivax (‘Chesson’ strain) is 0.5 mg/kg (30 mg in an adult) daily for 14 days in G6PD normal individuals. Data from G6PD deficient Cambodian airmen showed that 15 mg of daily primaquine resulted in mean fall in Hb of 3 g/dl on Day 7. Their G6PD activities were generally between 4–11% of normal; thus, most had severe class II G6PDd [4]. This fall would be higher in vivax infected patients treated with the same dose of PQ. G6PDd vivax infected patients are also at risk of haemolysis if they receive the recommended, 0.75 mg/kg weekly dose of primaquine for 8 doses. Data from a recently conducted safety *Plasmodium vivax* study on primaquine (Kheng S, Cambodian National Malaria Control Programme, unpublished data) suggest weekly primaquine (0.75 mg/kg) could be used under medical supervision in G6PDd patients.

Although, the evidence for the reduction of mosquito infectivity using the newly recommended low dose primaquine (0.25 mg/kg) in falciparum infected patients for transmission blocking have been recently reviewed by White and colleagues [2], data on primaquine safety in G6PDd individuals with falciparum malaria remain rare. There is a broad consensus on the needed to conduct prospective studies to confirm the safety of a single dose of primaquine as a gametocytocide together with ACT especially in Cambodia where artemisinin resistant falciparum parasites are circulating [33]. To address this question, a study is planned in Cambodia to evaluate its safety and to explore the applicability of the CareStart G6PD RDT (if necessary) as a PoC diagnostic to identify those patients who might suffer from significant haemolysis. For the CareStart G6PD RDT to efficiently work in real-life conditions, it was also critical to assess whether capillary samples performed equally well compared to the venous blood samples, as capillary samples are taken for slide preparation and malaria RDTs by health workers. Concordances between both capillary/venous blood samples were excellent. Only one discordance, with venous blood was found in a subject whose G6PD activity was 6.4 UI/g Hb (∼53% of normal G6PD enzyme activity). Similarly, only one white cell depleted venous sample gave a discordant result.

Although the performance of the CareStart G6PD RDT was assessed in the field, it was conducted by a research team. Therefore, there is a need to evaluate the test characteristics of the CareStart G6PD RDT by the users of this PoC, such as health facility staff and village malaria workers (VMWs). They are the malaria elimination “front line” in Cambodia and shoulder the responsibility for diagnosis, treatment, follow up and data recording. To accelerate the roll out of the CareStart G6PD RDT use and PQ in Cambodia, further evaluations have already been initiated to assess the operational challenges and programmatic usefulness of the tests when implemented by health workers in the field.

## Supporting Information

S1 Data **Data base listing all variables assessed in the study by individual.**
(XLSX)
